# Influence of the Size and Type of Pores on Brick Resistance to Freeze-Thaw Cycles

**DOI:** 10.3390/ma13173717

**Published:** 2020-08-22

**Authors:** Ivanka Netinger Grubeša, Martina Vračević, Vilma Ducman, Berislav Marković, Imre Szenti, Ákos Kukovecz

**Affiliations:** 1 Faculty of Civil Engineering and Architecture Osijek, Josip Juraj Strossmayer University of Osijek, Vladimira Preloga 3, 31000 Osijek, Croatia; 2 Institute IGH, Janka Rakuše 1, 10000 Zagreb, Croatia; 3 Slovenian National Building and Civil Engineering Institute, Dimičeva Ulica 12, 1000 Ljubljana, Slovenia; vilma.ducman@zag.si; 4 Department of Chemistry, Josip Juraj Strossmayer University of Osijek, Ulica Cara Hadrijana 8/A, 31000 Osijek, Croatia; bmarkovi@kemija.unios.hr; 5 Interdisciplinary Excellence Centre, Department of Applied and Environmental Chemistry, University of Szeged, H-6720, Rerrich Béla tér 1, 6720 Szeged, Hungary; szentiimre@gmail.com (I.S.); kakos@chem.u-szeged.hu (Á.K.)

**Keywords:** clay bricks, resistance to freeze-thaw cycles, compressive strength, MIP, micro-CT, Maage factor

## Abstract

This paper estimates the frost resistance of bricks using the ratio of compressive strength before freezing to compressive strength after freezing to describe the damage degree of bricks being exposed to freeze-thaw cycles. In an effort to find the ratio that clearly distinguishes resistant bricks from non-resistant bricks, the authors attempted to establish the correlation between the ratio and Maage factor as a recognized model for assessing brick resistance. To clarify the degree of damage of individual bricks, the pore size distribution has been investigated by means of mercury porosimetry. Additionally, micro computed X-ray tomography (micro-CT) has been employed to define the influence of the type of pores (open or closed) and their connectivity on the frost resistance of bricks. According to the results, it can be concluded that there is a good correlation between the Maage factor and the ratio of pre- to post-freeze-thaw cycle compressive strengths, and that the latter ratio strongly correlates with the percentage of large pores (≥3 mm) in the brick. If such a correlation could be confirmed in a larger sample, then the ratio of pre- to post-freeze-thaw cycle compressive strengths could be used as a new method for assessing brick resistance to freeze-thaw cycles and it would be possible to determine the minimum percentage of large pores required to ensure the overall resistance of brick to freeze-thaw conditions. The complexity of the problem is, however, evidenced by the fact that no clear connection between the type (open versus closed) or connectivity of pores and the frost resistance of bricks could be revealed by micro-CT.

## 1. Introduction

One of the main demands of external bricks or brick wall elements used as building materials is durability. Degradation of brick properties is primarily caused by salt crystallization and the cyclic freezing and thawing of water in the material [1,2,3,4]. During freezing, the expansion of ice may cause the development of straining inside the material. If there is no space to accommodate the expansion and the incurred straining exceeds the strength of the brick, then damage and/or cracks will occur.

European regulations stipulate that the frost resistance of bricks be evaluated according to CEN/TS 772-22 [5], where wall samples are directly exposed to freeze-thaw cycles. Global literature, however, additionally mentions various other indirect procedures and limits/critical values for each procedure in order to grade the resistance of bricks to freeze-thaw cycles. Canadian and American standards, for example, use compressive strength, boiling absorption, the saturation coefficient and water absorption to grade the resistance of bricks to freeze-thaw cycles [4,6]. Another highly acknowledged indirect procedure to predict the resistance of bricks to freeze-thaw cycles is the Maage factor [7,8,9,10]. The Maage factor is a statistical model based on experimental results, and it includes two main variables: the total volume of pores (PV) and the proportion of pores of a certain diameter, i.e., pores larger than 3 µm (P3), that are not fully saturated due to the meniscus effect. If there is a certain amount of space available in larger pores (larger than 3 µm), this can accommodate the pressure arising from ice formation. Maage states the following equation to obtain a coefficient of brick resistance to freeze-thaw cycles (Fc): Fc = 3.2 × PV + 2.4 × P3. The classification of results is as follows: Fc > 70—high probability that the material will be resistant to freeze-thaw cycles under severe climatic conditions; 55 < Fc < 70—uncertain zone of freeze-thaw resistance; and Fc < 55—low probability that the material will be resistant to freeze-thaw cycles under severe climatic conditions.

Alongside this simpler model, there are two modified models designed by Koroth [1]. Koroth has, in one model, connected the total volume of pores in the brick with the boiling absorption, and, in the second one, connected the proportion of pores larger than 3 µm in the brick with the boiling absorption, water absorption during a 1-h period and capillary absorption during exposure of the brick to water over a 4-h period. Conversely, Vincenzini [10] uses the critical radius of pores, F90, which is considered a 90% fractal, as a parameter to classify brick elements as resistant or non-resistant, while Franke and Bentrup [10] take the median radius of pores, F50. Litvan [11] estimates the resistance of bricks to freeze-thaw cycles based on the specific surface area of the pores, while Robinson [1,10] developed a coefficient based on the compressive strength, water absorption parameters and saturation coefficient. Arnott [10] defines the durability coefficients based on the strength and visual damage. Nakamura [10] developed three durability coefficients, one based on the physical properties of the brick elements, the second based on the specific volume of pores and a third based on a combination of the specific volume of pores and their distribution in the brick.

Each of the previously named procedures defines limit values where a brick is considered to be either resistant or non-resistant to freeze-thaw cycles, as well as the reliability of the method. Fagerlund [12] proved in his research that, if a brick sample is burdened by moisture under the critical point of saturation (“Scrit”), the same brick can be exposed to freeze cycles hundreds and thousands of times without any measurable damage. The point of saturation, Scrit, is reached when all of the open pores are saturated with moisture [12].

Consulting the previously identified reference literature, it can be seen that most of the authors consider porosity and the structure of pores to have a big influence on the durability properties of clay wall elements. Various research [1,6,7,8,9,10,11,13,14,15,16,17,18] suggests that the size of pores and the distance between pores significantly influence the properties of durability. According to Nakamura [1], pores with a diameter under 0.2 µm are not desirable for properties of durability. Arnott [1] claims that pores larger than 1–3 µm are desirable for durable properties of a clay product. According to Koroth and Maage, the estimation of the freezing durability is based not only on the total volume but also on the number of pores larger than 3 µm, which are easily filled and emptied of water and as such improve the durability properties of the brick [1,7,8,9,10].

In order to assess a clay product′s resistance to freeze/thaw cycles, researchers have been studying the changes of the following properties during freeze/thaw cycles: the surface appearance of the specimens [4,19,20,21], flexural strength and toughness [20], compressive strength and dynamic modulus of elasticity [22], propagation speed of ultrasonic waves through specimens [18,22], weight of specimens [21,22,23,24] and structure of pores [20].

It is evident from the literature cited in the previous paragraph that researchers rarely evaluate the resistance of bricks to freeze-thaw cycles by studying changes in compressive strength. Therefore, the authors of this paper decided to focus precisely on this brick property. The degree of brick damage experienced during the freeze-thaw cycles is observed here through the ratio of compressive strength before freezing to compressive strength after freezing. In an effort to find the ratio that clearly distinguishes resistant from non-resistant bricks, the authors attempted to establish the correlation between the ratio and the Maage factor as a recognized model for assessing brick resistance. To clarify the degree of damage of individual bricks, the pore size distribution has been investigated by means of mercury porosimetry. Additionally, micro computed X-ray tomography (micro-CT) has been employed to define the influence of the type of pores (open or closed) and their connectivity on the frost resistance of bricks.

## 2. Materials and Methods

### Description of Testing Methods

Eight series of commercially available factory-made bricks were sampled from building material depots in Croatia, Bosnia and Herzegovina, and Serbia, named here as S1–S8. Although European regulations test the resistance of bricks to freeze-thaw cycles according to CEN/TS 772-22 [5], the direct resistance of bricks to freeze-thaw cycles was tested here according to the standard HRN B.D8.011 [25] on four sets of bricks (one set is comprised of five bricks) within each brick series. Under the chosen standard, the samples were saturated with water and exposed to temperatures of −20 ± 2 °C for four hours in a climate chamber. The samples were subsequently submerged in water at a temperature of +15–20 °C, also for four hours. This cycle was repeated 25 times, and the brick was considered durable to freeze-thaw cycles if there were no signs of damage in any of the examined samples after 25 cycles of freezing and then defrosting in water. Both of the above assessment methods yield a solely qualitative grade for brick resistance to freeze-thaw cycles. Unlike in CEN/TS 772-22 [5], where brick resistance is tested by looking at a brick as part of a wall, in HRN B.D8.011 [25] the resistance of a brick is viewed by looking at the brick as a test unit and is thus more severe. After the bricks were exposed to freeze-thaw cycles according to HRN B.D8.011 [25], their compressive strength was determined, and the results were compared to the compressive strength of bricks from the same series which had not been exposed to the freeze-thaw cycles. In this way, a ratio between the compressive strengths before and after the freeze-thaw cycles was acquired as a quantitative indicator of the brick resistance to freeze-thaw behavior. The compressive strength before and after freezing was measured according to EN 772-1 [26] on ten brick samples (one brick sample was comprised of two brick units). Before testing, the surface of all specimens was prepared by grinding until the requirement for planeness and parallelism given in EN 772-1 [26] was achieved, and the specimens were conditioned (dried) at 105 ± 5 °C to a constant mass.

The structure of pores, their distribution, total porosity, the median radius of pores and the total pore volume (PV) of the bricks was determined using a Micromeritics AutoPore IV 9500 mercury intrusion porosimeter (MIP, Micromeritics, Norcross, GA, USA). For this test, fragments of brick with dimensions of approximately 1 cm^3^ were cut from each sample, dried for 24 h at 70 °C, degassed and then tested with mercury. Under high pressure (from 0 to 318 MPa), Hg penetrates into porous samples, and this gives information about the total open porosity as well as the pore size distribution. All the parameters determined by MIP were measured on three samples of each brick series.

The distribution of pores in the samples was also obtained via X-ray micro-computed tomography analysis, conducted using a Bruker Skyscan 2211 unit. Samples (Bruker, Kontich, Belgium) with a dimension of ~2.0 × 2.0 × 2.0 mm^3^ were scanned using an 11 Mp cooled CCD (charge coupled device) camera using a source voltage of 100 kV, a source current of 400 μA and an exposure time of 300 ms. The voxel size of this dataset was 0.4 × 0.4 × 0.4 μm. NRecon reconstruction software ((version 1.7.3.0)) was used to reconstruct the projected images with a pixel size of 4032 × 2688, and CTan (version 1.18) and CTvol (version 2.0) software were used to represent the 3D models. This device scopes pores sized 2–100 µm. The parameters determined by X-ray micro-computed tomography were obtained by measuring each type of brick sample three times, and the calculations were performed on several different volumes of interest (VOI). Example of cross-sectional slice as measured by micro-CT and the corresponding processed binary image used in the quantitative image analysis are given in Figure S1 of Supplementary materials.

## 3. Results

### 3.1. Assessment of Frost Resistance Using the Direct Method

**Figure 1:**
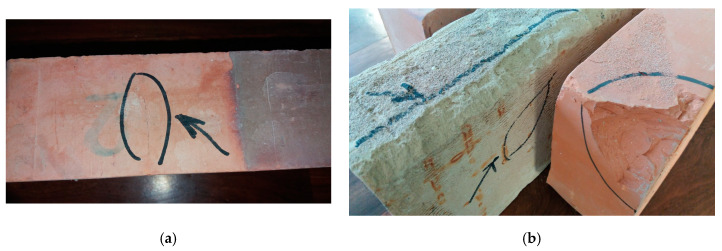
Typical appearance of non-resistant bricks after 25 freeze-thaw cycles, (**a**) damage in the form of cracking; (**b**) damage in the form of delamination.

Seven out of the eight brick series exposed to 25 freeze-thaw cycles experienced some form of damage, either cracking or delamination. The typical appearance of the brick samples after their exposure to the freeze-thaw cycles is shown in Figure 1a,b.

In accordance with HRN B.D8.01, a brick is deemed to be resistant to freeze-thaw cycles if, after 25 cycles of freezing at −20 °C followed by thawing in water, no sample show signs of damage. According to these criteria, only the S7 brick was shown to be resistant to freeze-thaw cycles, with the rest of the bricks being classified as non-resistant. The compressive strength of the bricks before and after freeze-thaw cycles with the corresponding standard deviation, as well as the ratio between the pre- and post-freeze-thaw cycle compressive strengths, are shown in Table 1. The compressive strengths (pre- and post-freezing) given here are the average values of ten individual measurements. The ratio of pre- to post-freezing compressive strengths was calculated by using the average values of the compressive strengths pre- to post-freezing.

### 3.2. Porosity and Pore Size Distribution

**Figure 2:**
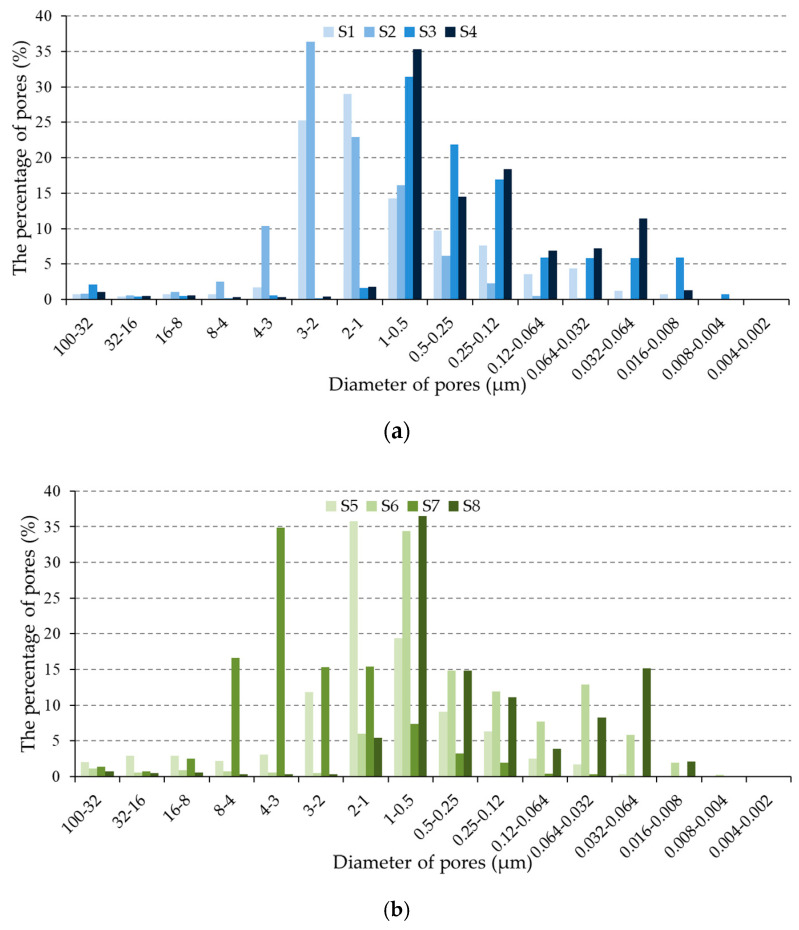
Pore size distribution in the brick series; (**a**) pore size distribution in brick series S1–S4; (**b**) pore size distribution in brick series S5–S8.

All the parameters determined by MIP were measured on three samples of each brick series, and considering the fact that the results of the three measurements were close to each other, the results given in Figure 2a,b and Table 2 present one sample, selected to be representative of a particular brick series. Figure 2a,b presents the pore size distribution in the brick series.

In the reference literature [1,14], pores smaller than 0.1 µm are considered to be small, while pores in the range of 0.1–1.0 μm are considered to be medium-sized pores. According to Maage [7,8,9,10] and some other authors [6,7,8,9,13,27,28], large pores are larger than 3 µm. In line with this, pores were categorized by size into groups of large, medium and small, as shown in Table 2. The median pore radius, total porosity and total pore volume, as measured by MIP, are also shown in Table 2.

### 3.3. Micro-CT Investigations

**Figure 3:**
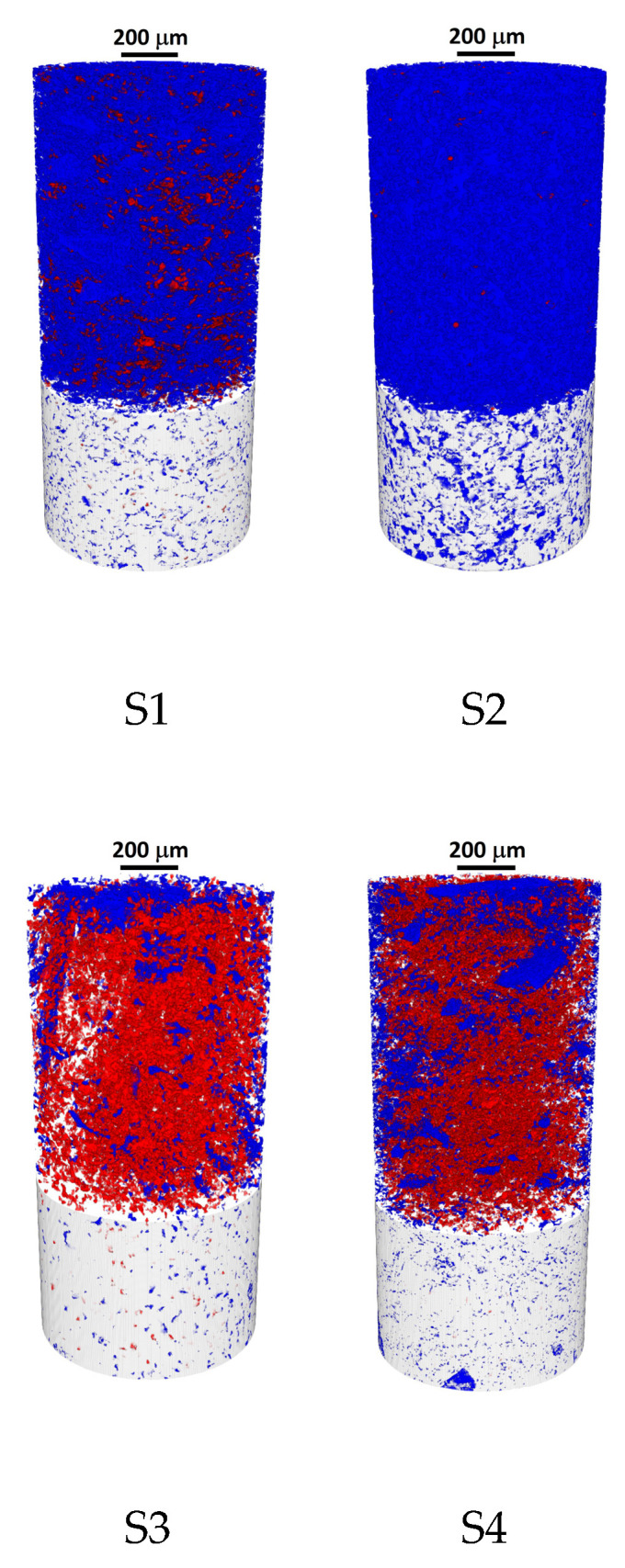

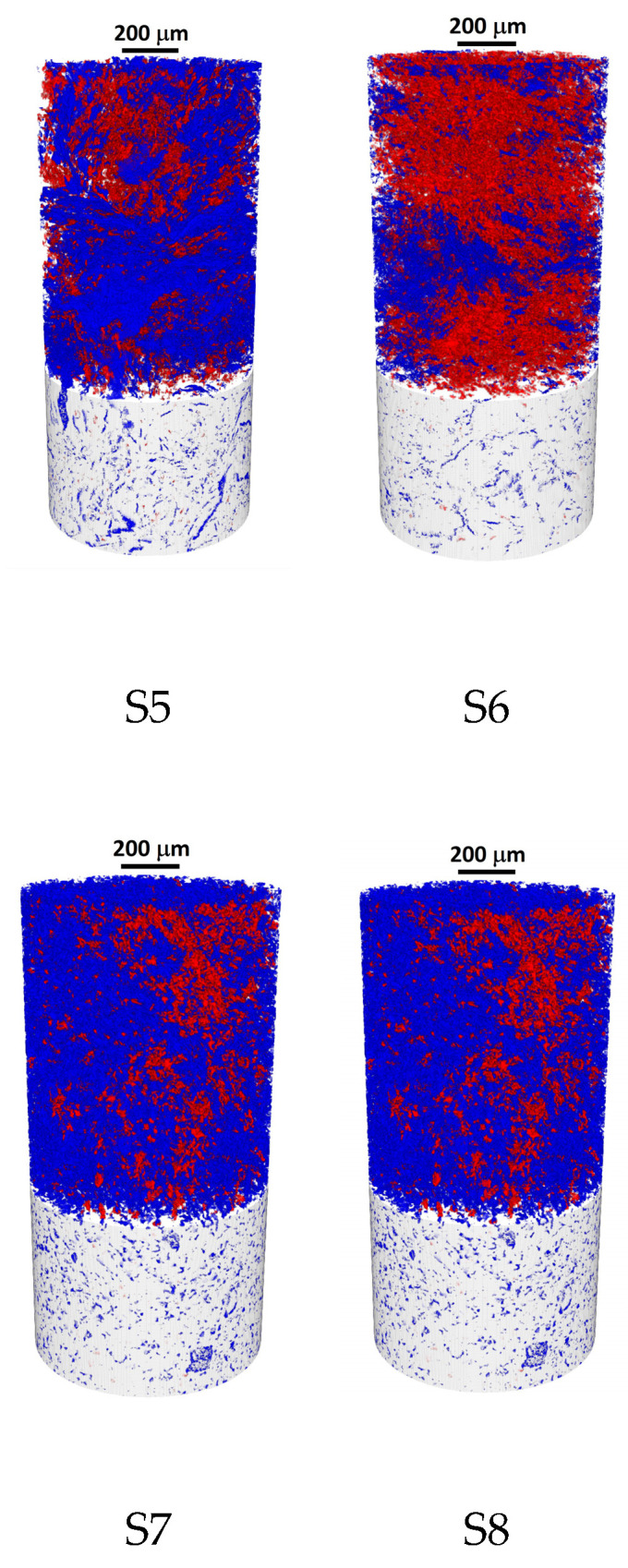
Characteristic micro-CT images of brick samples illustrating the quantitative differences reported in Table 3 (closed pores are marked in red, open pores in blue and the matrix is colored grey).

The applicability of micro-CT to freeze-thaw resistance analysis in mortars has recently been demonstrated [29]. Here, this method has been extended to the characterization of bricks, with the results being shown in Figure 3 and tabulated in Table 3. Open cylindrical volumes of interest (VOIs), used for the quantitative analysis of reconstructed images (Figure 3), were individually optimized for each sample to ensure the optimal representation of the heterogeneous pore structure in the VOI-based calculations. Closed pores are marked here in red, open pores in blue, and the matrix is colored grey. It is well-observable that the samples exhibit considerable variety in both the percentage and the spatial distribution of open and closed pores. The open/closed pore ratio ranges from 0.25 (S8) to 136 (S2). This factor of a ×500 difference is significantly higher than the total porosity percentage range, which barely spans a factor of ×6 (4.18% in S4 vs. 23.28% in S2). This indicates that pore connectivity is a complex characteristic of the samples that can only be interpreted in a three-dimensional context, as made available by micro-CT. It is worth noting that the trends observable from these micro-CT studies are in good qualitative agreement with the MIP results discussed below.

## 4. Discussion

The results of the brick tests regarding their resistance to freeze-thaw cycles obtained by HRN B.D8.011, the estimation based on the ratio of compression strengths after and before freezing, and the estimation based on the Maage factor are summarized in Table 4.

**Figure 4:**
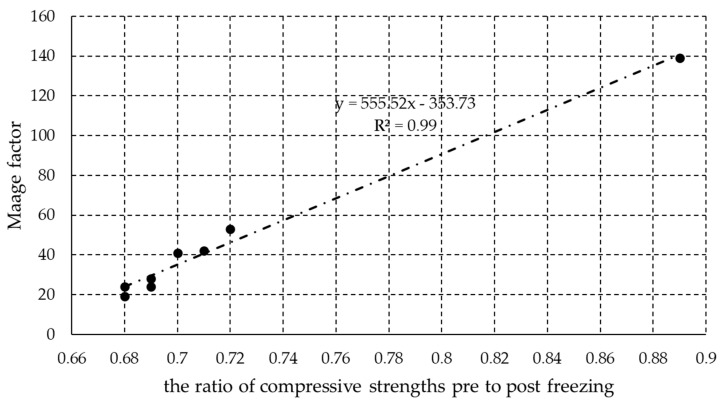
Correlation between the Maage factor and the ratio of pre- to post-freezing compressive strengths.

Figure 4 shows the relationship between the Maage factor and the ratio of the compressive strength before freezing to that after freezing for the series of bricks observed in the present study. Mallidi [30], who has surveyed numerous studies focused on the parameters influencing frost resistance, concluded that the Maage factor is one the most reliable indices for predicting the durability of bricks. However, for such an assessment, mercury intrusion porosimetry is necessary. Considering the limited availability of this method, there is a need to find links between the pore and absorption characteristics of bricks, or a way to evaluate durability in other terms, for example as a ratio of the pre- to post-freezing compressive strengths. A good relation between the Maage factor and the ratio of the pre- to post-freezing cycle compressive strengths was observed in the case of the machine-made bricks in [31]. In Figure 4, it can be seen that these two variables (Maage factor and the ratio of pre- to post-freezing compressive strengths) are strongly correlated, as seen by a high linear fit R2 value of 0.99. The equation describing their relationship is y = 555.52 × x − 353.73, where x is the ratio of the compressive strength before freezing to that after freezing and y is the Maage factor. If a value of 70, which is the lower limit of the Maage factor at which bricks are considered to be resistant to freeze-thaw cycles, is introduced into this equation, one can calculate that the ratio of the compressive strength before to that after freezing must be at least 0.76 in order for bricks to be considered resistant to freeze-thaw conditions. If the ratio of the pre- to post-freezing compressive strengths is to be used as an indicator of the resistance of brick to freeze-thaw cycles, then a larger database will be needed in order to firmly identify classification limits. The authors of this paper will focus their future efforts in this direction.

Furthermore, in an effort to find an answer to the effects of the pore size and type (open or closed), as well as their interconnection, on the resistance of brick to freeze-thaw cycles, these parameters are again extracted in Table 5.

Table 5 shows that the ratios of the pre- to post-freezing compressive strengths correlate very well with the percentage of large pores.

**Figure 5:**
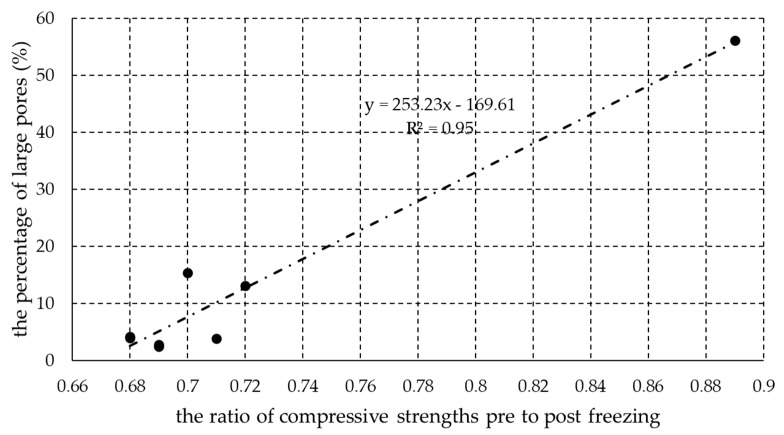
Correlation between the percentage of large pores and the ratio of pre- to post-freezing compressive strengths.

In Figure 5, it can be seen that these two variables are linearly correlated, with an R2 value of 0.95. The equation describing their relationship is y = 253.23 × x − 169.61, where x is the ratio of the compressive strength before to that after freezing, and y is the percentage of large pores. In the previous step, it was suggested that a value of 0.76 could be used as a lower limit for the pre- to post-freezing compressive strength ratio in order to classify a brick as being freeze-thaw resistant. Taking this value forward to the second linear equation, we can further estimate that the percentage of large pores should be at least 23% for the brick to be considered as being resistant to freeze-thaw cycles.

The total porosity of bricks, as determined by tomography, is lower than the porosity determined by MIP. This is to be expected given a certain limitation of tomography, which, in the present case, can only detect pores bigger than 2 μm, whereas MIP measurements can detect pores as small as 0.0055 µm, consequently giving a higher porosity result [32]. The tomography results presented here indicate no clear connection between either the type of pores (open or closed) or their connectivity and the frost resistance of bricks. This is readily explained by the three orders of magnitude of difference in the lowest pore diameter range, as measured by MIP and micro-CT. Work is in progress in our laboratory to address this complex problem from another aspect, e.g., via a micro-CT spatial inhomogeneity analysis.

## 5. Conclusions

This paper estimates the frost resistance of bricks using the ratio of the compressive strength before freezing to the compressive strength after freezing to describe the damage degree of bricks being exposed to freeze-thaw cycles. In an effort to find the ratio that clearly distinguishes resistant from non-resistant bricks, the authors attempted to establish the correlation between the ratio and the Maage factor as a recognized model for assessing brick resistance. To clarify the degree of damage of individual bricks, the pore size distribution was investigated by means of mercury porosimetry. Additionally, micro computed X-ray tomography (micro-CT) was employed to define the influence of the type of pores (open or closed) and their connectivity on the frost resistance of bricks. By analyzing the research results, the following conclusions could be made:

- A strong relationship was observed between the Maage factor and the ratio of the compressive strength before to that after freeze-thaw cycles. If such a correlation is confirmed on a larger sample, the ratio of the compressive strength before to that after freeze-thaw cycles could be used as a new method for assessing brick resistance to freezing and thawing.
- The ratios of the compressive strength before to that after freezing correlate very well with the percentage of large pores, meaning that further research in this direction might be able to determine the minimum percentage of large (≥3 μm) pores required in order to ensure the overall resistance of brick to freeze-thaw cycles.
- In this research, no clear connection was observed between the type of pores (open or closed) or their connectivity and the frost resistance of bricks.

## Figures and Tables

**Table 1:** Compressive strength of bricks before and after freezing with the corresponding standard deviation and the ratio of compressive strengths pre- to post-freezing.

Brick Series/Property Tested	S1	S2	S3	S4	S5	S6	S7	S8
Normative compressive strength before freezing (N/mm^2^)	17.7 ± 0.4	8.0 ± 0.2	28.7 ± 0.6	28.4 ± 0.4	15.2 ± 0.2	27.9 ± 0.3	27.7 ± 0.3	27.8 ± 0.3
Normative compressive strength after freezing (N/mm^2^)	12.1 ± 0.3	5.6 ± 0.1	20.4 ± 0.4	19.70 ± 0.3	10.9 ± 0.2	18.9 ± 0.3	24.6 ± 0.3	19.13 ± 0.3
The ratio of compressive strength before freezing to compressive strength after freezing	0.68	0.70	0.71	0.69	0.72	0.68	0.89	0.69

**Table 2:** Proportion of pores of a given size, total porosity, median pore radius and total pore volume in the bricks.

Property/ Brick Series	Proportion of Pores of a Given Size (%)			Total Porosity (%)	Median Pore Radius (mm)	Total Volume of Pores, PV (cm^3^/g)
	Large	Medium	Small			
S1	4.2	85.9	9.9	37.7	0.25	232.5
S2	15.4	83.9	0.7	46.11	1.01	370.5
S3	3.8	71.9	24.3	30.07	0.26	168.5
S4	2.8	70.4	26.8	30.77	0.08	171.2
S5	13.1	82.4	4.5	28.86	0.51	154.3
S6	3.9	67.6	28.5	32.99	0.09	183.2
S7	56.1	43.2	0.7	34.42	1.45	207.5
S8	2.4	68.1	29.5	32.34	0.05	813.3

**Table 3:** The percentage of open pores, the percentage of closed pores and the interconnection of pores.

Brick Series/Property Tested	S1	S2	S3	S4	S5	S6	S7	S8
The percentage of open pores (%)	11.95	23.11	1.42	1.13	8.51	2.52	16.01	0.92
The percentage of closed pores (%)	0.85	0.17	2.56	3.05	1.43	2.34	0.41	3.61
The total percentage of pores (%)	12.8	23.28	3.98	4.18	9.94	4.86	16.42	4.53
Interconnection of pores (mm^−3^)	188,227	268,300	30,930	108,688	162,121	131,387	155,587	59,451

**Table 4:** Summarized results of the methods applied to test brick resistance to freeze-thaw cycles.

Brick Series/Property Tested	S1	S2	S3	S4	S5	S6	S7	S8
Resistance according to HRN B.D8.011	Non-resistant	Non-resistant	Non-resistant	Non-resistant	Non-resistant	Non-resistant	Resistant	Non-resistant
The ratio of pre- to post-freezing compressive strengths	0.68	0.70	0.71	0.69	0.72	0.68	0.89	0.69
The Maage factor; estimation of resistance based on the Maage factor	24.0—low probability of resistance	41.0—low probability of resistance	42.0—low probability of resistance	28.0—low probability of resistance	53.0—low probability of resistance	19.0—low probability of resistance	139.0—high probability of resistance	24.0—low probability of resistance

**Table 5:** Summarized results of the brick resistance to freeze-thaw cycles, porosimetry and micro-CT.

Brick Series/Property Tested		S1	S2	S3	S4	S5	S6	S7	S8
The ratio of the compressive strength before to that after freezing		0.68	0.70	0.71	0.69	0.72	0.68	0.89	0.69
**MIP results**	The percentage of large pores (%)	4.2	15.4	3.8	2.8	13.1	3.9	56.1	2.4
	The percentage of medium pores (%)	85.9	83.9	71.9	70.4	82.4	67.6	43.2	68.1
	The percentage of small pores (%)	9.9	0.7	24.3	26.8	4.5	28.5	0.7	29.5
	The total percentage of pores (%)	37.7	46.11	30.07	30.77	28.86	32.99	34.42	32.34
	Median radius of the pores (mm)	0.25	1.01	0.26	0.08	0.51	0.09	1.45	0.05
**micro-CT results**	The percentage of open pores (%)	11.95	23.11	1.42	1.13	8.51	2.52	16.01	0.92
	The percentage of closed pores (%)	0.85	0.17	2.56	3.05	1.43	2.34	0.41	3.61
	The total percentage of pores (%)	12.8	23.28	3.98	4.18	9.94	4.86	16.42	4.53
	Interconnection of pores (mm^−3^)	188,227	268,300	30,930	108,688	162,121	131,387	155,587	59,451

## Supplementary Materials

The following are available online at https://www.mdpi.com/1996-1944/13/17/3717/s1, Figure S1. Example of cross-sectional slice as measured by micro-CT (**a**), and the corresponding processed binary image (**b**) used in the quantitative image analysis.
