# Supplementary material for: Influence of the Size and Type of Pores on Brick Resistance to Freeze-Thaw Cycles
Source: Materials (Basel). 2020 Aug 22;13(17):3717. doi: 10.3390/ma13173717 (PMC7503822; doi:10.3390/ma13173717)
Supplement: Supplementary file 1 [file materials-13-03717-s001.pdf]

materials

Supplementary Materials

Influence of the Size and Type of Pores on Brick Resistance to Freeze-Thaw Cycles

Ivanka Netinger Grubeša [1,*], Martina Vračević [2,*], Vilma Ducman [3], Berislav Marković [4], Imre Szenti [5] and Ákos Kukovecz [5]

1 Faculty of Civil Engineering and Architecture Osijek, Josip Juraj Strossmayer University of Osijek, Vladimira Preloga 3, 31000 Osijek, Croatia
2 Institute IGH, Janka Rakuše 1, 10000 Zagreb, Croatia
3 Slovenian National Building and Civil Engineering Institute, Dimičeva Ulica 12, 1000 Ljubljana, Slovenia; vilma.ducman@zag.si
4 Department of Chemistry, Josip Juraj Strossmayer University of Osijek, Ulica Cara Hadrijana 8/A, 31000 Osijek, Croatia; bmarkovi@kemija.unios.hr
5 Interdisciplinary Excellence Centre, Department of Applied and Environmental Chemistry, University of Szeged, H-6720, Rerrich Béla tér 1, 6720 Szeged, Hungary; szentiimre@gmail.com (I.S.); kakos@chem.u-szeged.hu (Á.K.)
* Correspondence: nivanka@gfos.hr (I.N.G.); martina.vracevic@igh.hr (M.V.)

Received: 29 June 2020; Accepted: 17 August 2020; Published: 22 August 2020

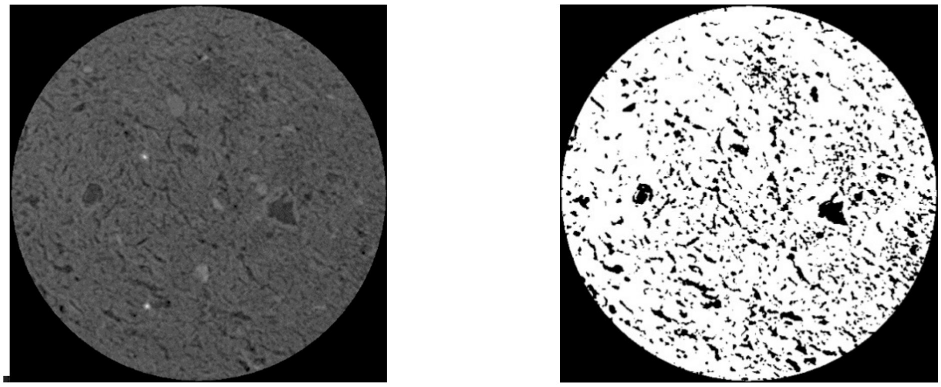

(**a**) (**b**)

Figure S1. Example of cross-sectional slice as measured by micro-CT (**a**), and the corresponding processed binary image (**b**) used in the quantitative image analysis.

© 2020 by the authors. Licensee MDPI, Basel, Switzerland. This article is an open access article distributed under the terms and conditions of the Creative Commons Attribution (CC BY) license (http://creativecommons.org/licenses/by/4.0/).
